# Relationship between cardiac magnetic resonance imaging parameters and pregnancy outcomes in women post mustard repair: a multi-center study

**DOI:** 10.1186/1532-429X-15-S1-P299

**Published:** 2013-01-30

**Authors:** Laura Jimenez Juan, Anne Marie Valente, Bernd J Wintersperger, Candice Silversides, Andrew M Crean, Jack M Colman, Elsie T Nguyen, Tal Geva, Rachel M Wald

**Affiliations:** 1 Radiology, Sunnybrook Health Sciences Centre, Toronto, ON, Canada; 2 Cardiology, Boston Children's Hospital, Boston, MA, USA; 3 Radiology, Toronto General Hospital, Toronto, ON, Canada; 4 Cardiology, Toronto General Hospital, Toronto, ON, Canada

## Background

Summary: To examine the relationship between cardiovascular magnetic resonance imaging (CMR) parameters and pregnancy outcomes post Mustard palliation.

Background: Impaired systemic ventricular function and presence of a systemic right ventricle (RV) are associated with adverse events in pregnancy. Women with transposition of the great arteries post atrial switch (Mustard procedure) are at risk of worsening arrhythmia and heart failure antenatally. Contemporary guidelines suggest that women with "more moderate systemic RV dysfunction" should be advised against pregnancy; however a threshold RV ejection fraction (EF) has not been defined as all studies to date have examined RV function using echocardiography alone. The CMR characteristics of women post Mustard palliation undergoing pregnancy and the relationship between CMR parameters and pregnancy outcomes have not yet been described.

## Methods

A total of 17 consecutive women post Mustard procedure seen at 2 tertiary care centers who had undergone CMR within 2 years of pregnancy were included. Parameters of ventricular function were assessed by a single experienced reader using steady-state free-precession cine CMR acquired in the short-axis orientation. Adverse cardiovascular events (sustained arrhythmia, heart failure, stroke, cardiac arrest and/or urgent cardiac intervention), obstetric complications (eclampsia, pre-term labour, hemorrhage) and fetal/neonatal events (stillbirth/death, prematurity, low birthweight, intensive care unit admission) were recorded.

## Results

Demographics and pregnancy outcomes are detailed in table 1. All women were asymptomatic (NYHA 1) at baseline. Cardiovascular events were observed in 3 women (3/17, 18%), obstetric complications were seen in 2 women (2/17, 12%), and fetal/neonatal events were seen in 3 infants (3/17, 18%)(table 1). Median gestational age at delivery and birthweight were 38 weeks (24-39 weeks) and 2770 g (2195-3720g), respectively. Median RV end diastolic volume (EDV) was 119 mL/m2 (85-214mL/m2), stroke volume was 75 mL (57-138mL) and RVEF was 38% (30-51%). RVEF was ≥35% in 10/17 (59%) and <35% in 7/17 (41%) women. Of those with RVEF<35%, 3/7 (43%) had a cardiovascular event. The median RVEF was 33% (32-34%) in those with a cardiovascular event compared with 38% (30-51%) in those without a cardiovascular event. There was a trend toward statistical significance for occurrence of cardiovascular events in women with RVEF <35% (p=0.051). Adverse obstetrical or fetal/neonatal events did not relate to decreased RVEF.

**Table 1:** Summary of demographic characteristics and outcomes

Maternal demographic data	Median (range)
Age at Mustard palliation (years)	2.2 (0.6-6)

Age at delivery (years)	31 (25-37)

Age at CMR (years)	32 (27-37)

Gravidity	n (%)

Gravida 1	5 (31%)

Gravida 2	3 (19%)

Gravida ≥ 3	8 (50%)

Cardiac anatomy	

Transposition of the great arteries with intact septum	14 (82%)

Transposition of the great arteries with ventricular septal defect	2 (12%)

Transposition of the great arteries with aortic coarctation	1 (6%)

Cardiovascular events in pregnancy	

Decline in NYHA classification	1 (6%)

Sustained tachyarrhythmia resulting in heart failure	1 (6%)

Cardiac arrest	1 (6%)

Obstetric events	

Premature rupture of membranes	1 (6%)

Placental abruption	1 (6%)

Fetal/neonetal events*	

Premature delivery < 37 weeks	3 (18%)

Admission to intensive care unit	4 (24%)

Death	1 (6%)

*events not mutually exclusive	

## Conclusions

Impaired RVEF, specifically RVEF <35% as determined by CMR, may be useful for stratification of risk for pregnancy-related cardiovascular complications in women post Mustard palliation. These preliminary findings require validation in a larger cohort of patients.

## Funding

None

